# Progressive modulation of resting-state brain activity during neurofeedback of positive-social emotion regulation networks

**DOI:** 10.1038/s41598-021-02079-4

**Published:** 2021-12-03

**Authors:** Marina Krylova, Stavros Skouras, Adeel Razi, Andrew A. Nicholson, Alexander Karner, David Steyrl, Olga Boukrina, Geraint Rees, Frank Scharnowski, Yury Koush

**Affiliations:** 1 Department of Psychiatry and Psychotherapy, Jena University Hospital, Jena, Germany; 2 Department of Biological and Medical Psychology, University of Bergen, Bergen, Norway; 3 Turner Institute for Brain and Mental Health, Monash University, Clayton, Australia; 4 Wellcome Centre for Human Neuroimaging, University College London, London, UK; 5 Department of Psychiatry and Behavioural Neurosciences, McMaster University, Hamilton, Canada; 6 Department of Cognition, Emotion, and Methods in Psychology, University of Vienna, Vienna, Austria; 7 Department of Psychiatry, Psychotherapy and Psychosomatics, Psychiatric Hospital, University of Zurich, Zurich, Switzerland; 8 Center for Stroke Rehabilitation Research, Kessler Foundation, West Orange, NJ USA; 9 Department of Physical Medicine and Rehabilitation, Rutgers-New Jersey Medical School, Newark, NJ USA; 10 Department of Radiology and Biomedical Imaging, Magnetic Resonance Research Center, Yale University, 300 Cedar Street, New Haven, CT 06519 USA

**Keywords:** Cognitive control, Prefrontal cortex, Amygdala, Consolidation

## Abstract

Neurofeedback allows for the self-regulation of brain circuits implicated in specific maladaptive behaviors, leading to persistent changes in brain activity and connectivity. Positive-social emotion regulation neurofeedback enhances emotion regulation capabilities, which is critical for reducing the severity of various psychiatric disorders. Training dorsomedial prefrontal cortex (dmPFC) to exert a top-down influence on bilateral amygdala during positive-social emotion regulation progressively (linearly) modulates connectivity within the trained network and induces positive mood. However, the processes during rest that interleave the neurofeedback training remain poorly understood. We hypothesized that short resting periods at the end of training sessions of positive-social emotion regulation neurofeedback would show alterations within emotion regulation and neurofeedback learning networks. We used complementary model-based and data-driven approaches to assess how resting-state connectivity relates to neurofeedback changes at the end of training sessions. In the experimental group, we found lower progressive dmPFC self-inhibition and an increase of connectivity in networks engaged in emotion regulation, neurofeedback learning, visuospatial processing, and memory. Our findings highlight a large-scale synergy between neurofeedback and resting-state brain activity and connectivity changes within the target network and beyond. This work contributes to our understanding of concomitant learning mechanisms post training and facilitates development of efficient neurofeedback training.

## Introduction

Resting-state functional magnetic resonance imaging (rs-fMRI) has sparked widespread interest after observation that spontaneous blood oxygenation level-dependent (BOLD) signal oscillations are organized into large-scale networks^1,2^. Self-regulation refers to the ability to manage one’s own emotional, cognitive and behavioral functions and can be learned through neurofeedback^3,4^. Neurofeedback is a form of training that enables individuals to learn voluntary self-regulation of brain circuits implicated in specific behavior or pathology^4^. Importantly, improvements in clinical symptoms are observed directly after neurofeedback training and persist for weeks^5^ or even months^6–10^ after the training. Neurofeedback is associated with post-training resting-state functional connectivity (rs-FC) modulations in target brain areas and beyond them, as detected immediately after neurofeedback training^9,11,12^, several days after training^13^ or weeks/months after training^14–16^. Alterations in rs-FC have been reported for different types of fMRI feedback types, specifically for activity^15,17,18^, functional connectivity^11,19^ and decoding^20^. In studies with emotion regulation neurofeedback, rs-FC modulations were also consistently observed after neurofeedback training between amygdala and the (pre)frontal–temporal-limbic areas^12,15,17,21^.

According to emotion regulation models, successful regulation is achieved through the modulation of bottom-up emotional responses in limbic areas by cognitive top-down processes originating in prefrontal cortices^22–27^. This concept has also been demonstrated during the regulation of positive emotions^15,28–30^. A comprehensive understanding of positive emotion upregulation in social situations (positive-social emotion upregulation) is critical as impairment of this ability may constitute a key factor contributing to the anhedonic and emotion dysregulation symptoms associated with depression and posttraumatic stress disorder^26,31–34^. Recently, it has been shown that positive-social emotion upregulation is associated with the direct influence of the prefrontal cortices on bilateral amygdala^30,35^. Consistently, positive-social emotion regulation based on effective connectivity neurofeedback enhances top-down emotion regulation capabilities and results in concomitant behavioral changes^29^. Effective connectivity was estimated using dynamic causal modeling (DCM) as a degree of a top-down network model dominance (from dorsomedial prefrontal cortex (dmPFC) onto bilateral amygdala) over a bottom-up one (from bilateral amygdala onto dmPFC). Neurofeedback-induced behavioral changes implied increases in valence ratings of emotional stimuli after as compared to before training.

Previously, training a bidirectional connectivity change between primary motor and lateral parietal cortices in healthy participants led to progressive rs-FC modulations of large networks across short resting-states at the beginning of the training sessions^19^. However, authors did not observe significant progressive rs-FC modulations between the trained areas, as well as their resting-state scans were not acquired right after the neurofeedback training. Similar resting-state scans were acquired in patients with borderline personality disorder, and progressive increases in amygdala-dlPFC rs-FC was observed during neurofeedback as a mechanism to induce amygdala downregulation^36^. Gradual recovery processes were observed during inbuilt rest of visual-spatial attention neurofeedback runs that consisted of interleaved neurofeedback and baseline conditions with 1 min rest epochs^37^, however progressive rs-FC modulations were not addressed. Thus, the role of rest in neurofeedback learning and concomitant resting-state neural modulations remains unexplored, where filling this knowledge gap could help understand learning mechanisms and improve design efficiency of neurofeedback training paradigms.

Here, we hypothesized that short resting-state periods at the end of positive-social emotion regulation neurofeedback training sessions would reflect progressive neural modulations of the trained network model (i.e., connectivity alterations between the dmPFC and bilateral amygdala)^29^ and networks engaged in emotion regulation and neurofeedback learning. To test this hypothesis, we employed a comprehensive and multidimensional approach to previously unpublished resting-state data from our positive-social emotion regulation neurofeedback study^29^. To exhaustively elucidate brain network resting-state activity and connectivity changes given the novelty of this study, we examined orthogonal, yet complimentary analyses. Five analyses were carried out, pertaining to (i) model-based effective connectivity of target brain network areas based on spectral DCM (spDCM), (ii) whole-brain data-driven spatiotemporal patterns of spontaneous brain activity based on tensorial Independent Component Analyses (tICA), (iii) whole-brain FC estimates based on the eigenvector centrality (EC), (iv) FC density (FCD), and (v) seed-based FC. Importantly, these measures provide unique information regarding various aspects of network dynamics. Specifically, by means of the spDCM^38–40^, we investigated whether learning positive-social emotion regulation using effective connectivity neurofeedback leads to progressive (linear) increase in resting-state effective connectivity within the trained network. By means of the data-driven tICA^41,42^, we investigated whether such neurofeedback training leads to a progressive increase of whole-brain spatiotemporal patterns of spontaneous resting-state activity. To examine whether neurofeedback training modulates emotion regulation and neurofeedback learning FC networks, conventional rs-FC analyses were performed based on voxel-wise Pearson correlations. We performed whole-brain EC analyses to reveal modulations of brain areas that are central within the small‐world network and strongly correlated with other nodes^43^. We performed whole-brain FCD analyses to identify modulations of brain areas with large long-range correlations. Finally, seed-based FC analyses unveiled modulations of resting-state dmPFC connectivity with the rest of the brain.

## Results

We first briefly describe neurofeedback task-related learning effects of the positive-social emotion regulation based on effective connectivity neurofeedback training, which were thoroughly reported and discussed in a previous publication^29^. We then continue with new results associated with intermediate rest periods in terms of the progressive (linear) increase of resting-state (i) network model-based effective connectivity, (ii) whole-brain data-driven spatiotemporal spontaneous activity and (iii) whole-brain functional connectomics.

### Neurofeedback task-related learning effects

**Figure 1:**
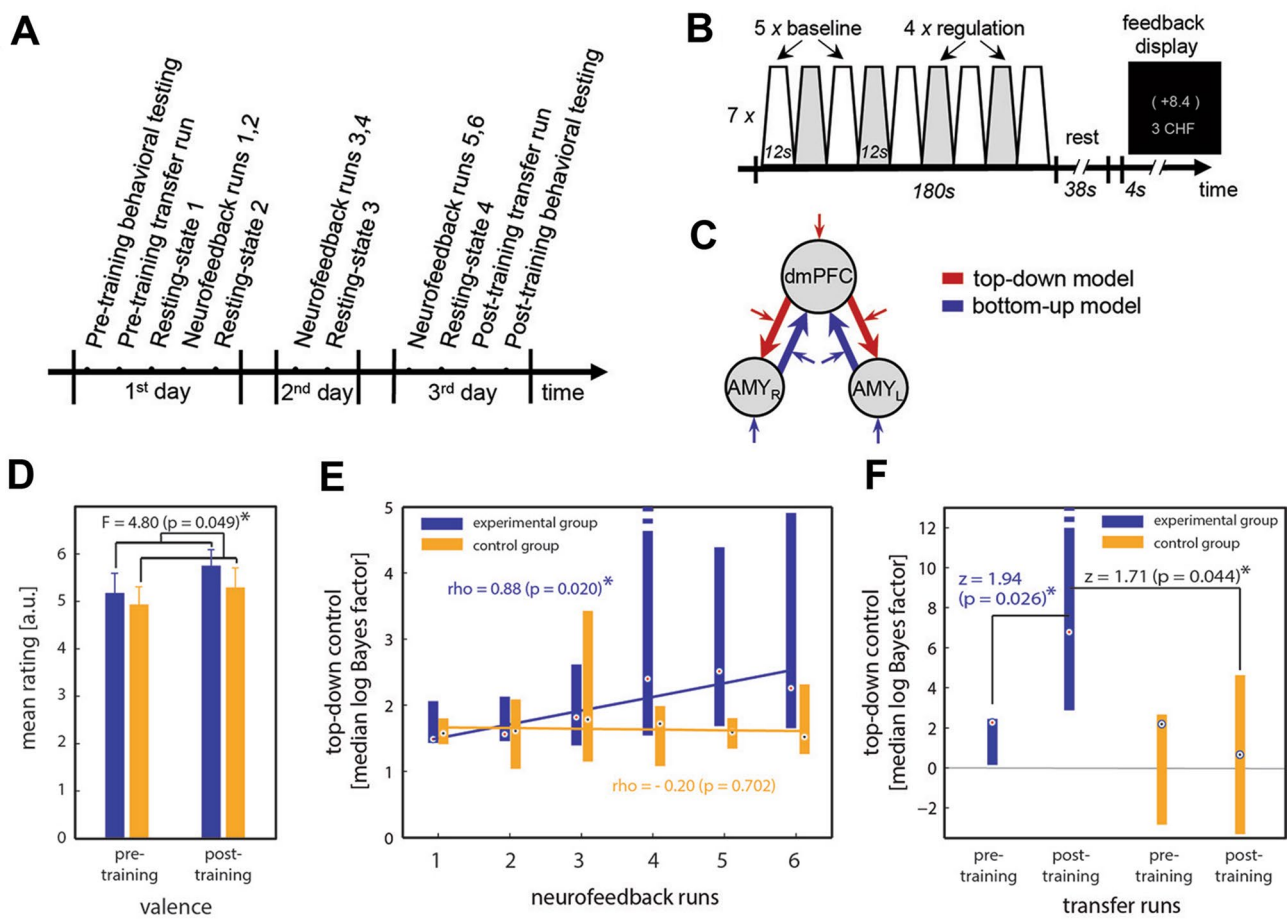
Neurofeedback study design and learning effects. (**A**) Participants in the experimental and control (sham) groups performed a 3-day study which included 3 neurofeedback training sessions with two ~ 17.5 min training runs per day, pre-/post-training behavioral tests, questionnaires and transfer runs, as well as four 6.1 min eyes-closed resting-state runs. (**B**) Each neurofeedback run consisted of 7 trials of four 12 s regulation blocks interleaved with five 12 s baseline blocks followed by a 38 s black-screen rest period and 4 s feedback presentation. During regulation blocks, moderately positive-social images were presented and participants were asked to control their positive emotions to maximize the feedback signal. During baseline blocks participants were asked to passively observe images of neutral objects. The feedback display consisted of a feedback value and cumulative monetary reward that had been earned until then. (**C**) Two opposite model architectures that represented top-down modulation from dmPFC onto bilateral amygdala (red), and bottom-up information flow from bilateral amygdala onto dmPFC (blue) were compared. The degree of dominance of the top-down model over the bottom-up model in terms of the logarithmic Bayes factor was used as a feedback signal. (**D**) The experimental but not the control group showed significantly more positive valence ratings of positive-social pictures after as compared to before training. (**E**) Participants in the experimental group showed a significant increase in top-down control with training and that was not present in the control group (two-tailed two-sample t-test on slopes, t(13) = 2.40, p = 0.03). (**F**) Learned control over emotion networks in transfer runs without feedback. *dmPFC* dorsomedial prefrontal cortex, *AMY* amygdala, *L* left, *R* right.

The multivariate analysis of variance (MANOVA) detected a main effect of group (experimental vs. control), given differences in valence and arousal ratings, and slopes of the learning curves as dependent variables [F(3,11) = 6.93, p = 0.007]^29^. The ANOVA on valence ratings revealed the main effect of group [Fig. 1D, F(1,12) = 4.80, p = 0.049], which was driven by an expected significant increase in valence ratings due to neurofeedback training in the experimental [one-tailed paired t-test, t(8) = 2.04, p = 0.038], but not in the control group [one-tailed paired t-test, t(5) = 1.33, p = 0.120]. In the experimental group, the effect size for valence ratings was large (Cohen’s d = 0.53). There was no effect of unequal group sizes on observed differences between experimental and control group behavior (for details, see Supplementary Information).

The slope of the learning curve indicated learning success via Pearson rho values, which indexed the linear relationship between logarithmic Bayes factors and neurofeedback training runs. Participants in the experimental group learned to increase the dominance of the top-down effective connectivity model compared to the bottom-up model (Fig. 1E; experimental group rho = 0.88, p = 0.020; control group rho =  − 0.20, p = 0.702; two-tailed two-sample t-test on slopes, t = 2.40, p = 0.032). In the transfer runs without neurofeedback, participants in the experimental group could control top-down connectivity after training significantly better than before (Fig. 1F: one-tailed Wilcoxon rank sum tests and z-statistics, experimental group: z = 1.94, p = 0.026; control group, z = 0.40, p = 0.345), leading to the higher dominance of the top-down model in the post-training transfer run (z = 1.71, p = 0.044). In the experimental group, the dmPFC was significantly more active in the post-training transfer run as compared to the pre-training transfer run (MNI coordinates [− 5/5 63/52 31/34], p < 0.05), while in the control group a significant increase in left amygdala activity was observed (MNI coordinates [− 25 5 − 16], p < 0.05). Learning control over top-down model increased activation in dmPFC (rho = 0.81, p = 0.049) and decreased activation in right amygdala (rho = − 0.81, p = 0.049) in the experimental group. In contrast, participants in the control group increased activation in left amygdala (rho = 0.86, p = 0.028).

### Resting-state network model-based effective connectivity by spDCM

**Figure 2:**
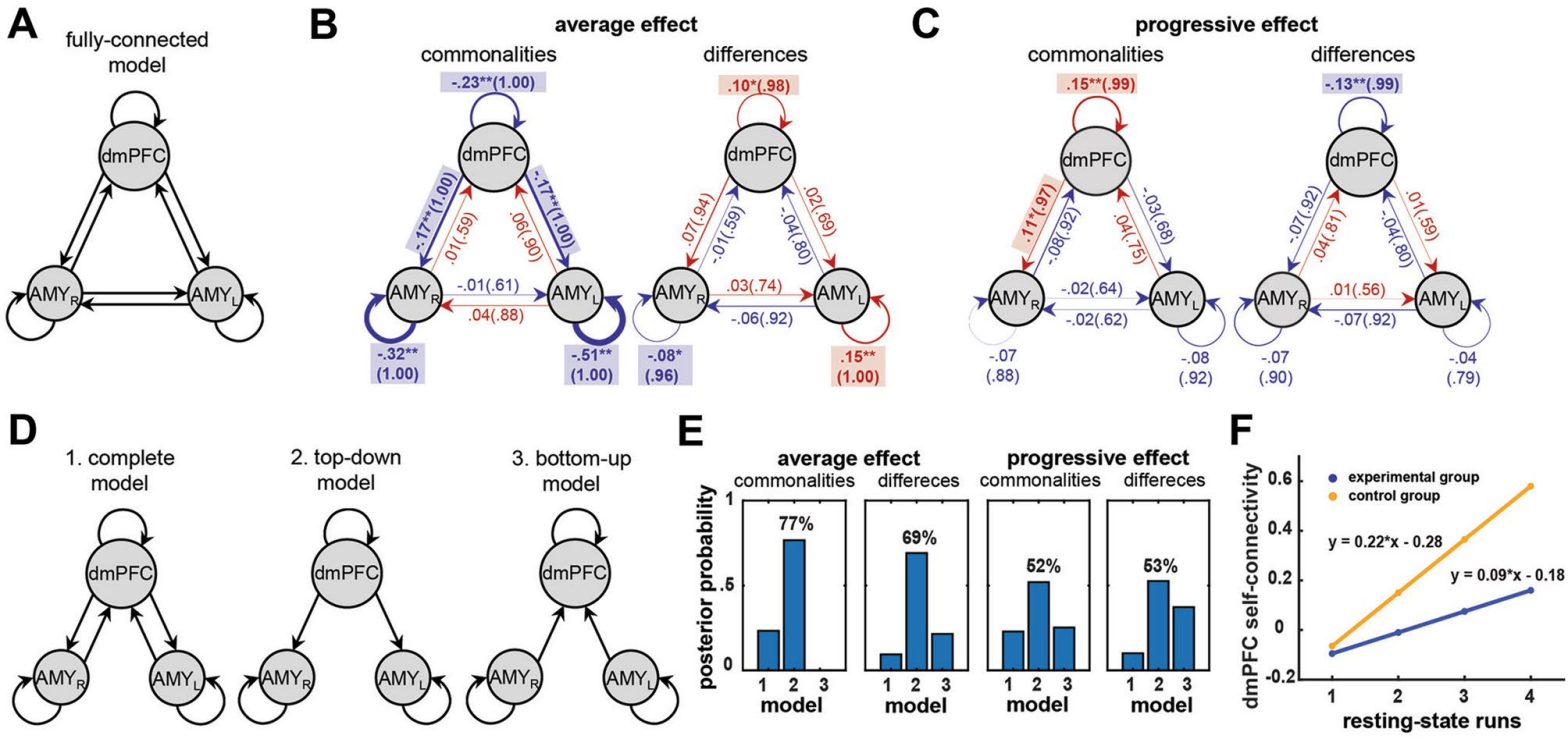
Resting-state effective connectivity associated with neurofeedback training. (**A**) A fully connected dmPFC and bilateral amygdala network architecture that was used to model progressive changes (over training runs) in resting-state effective connectivity. The reciprocal connections between ROIs and their self-connections are indicated with arrows. (**B**) The average effect of neurofeedback training across resting-state runs for commonalities (i.e., the mean connectivity across both groups) and differences between experimental and control groups. Note that DCM parameters per group can be derived using average and difference equations (panel **F**). (**C**) The progressive effect for commonalities and differences between experimental and control groups. Progressive self-inhibition of dmPFC was significantly lower in the experimental group. Negative values are depicted in blue, positive in red. Connectivity strength is illustrated by the proportional arrow thickness with posterior probability (Pp) in brackets. Significant connectivity parameters are indicated with shaded rectangles and bold font. Asterisks denote statistical significance (** for Pp > .99, * for Pp > 0.95). (**D**) Network architecture of target models. Fully-connected, top-down and bottom-up DCM models without direct connections between bilateral amygdala were selected to find the most likely resting-state network model associated with learning control of top-down self-regulation over bottom-up self-regulation (Fig. 1C). (**E**) Posterior probability of models for commonalities and differences across experimental and control groups. The top-down model explained average and progressive effects remarkably better than other models and was more evident in the experimental than in the control group. (**F**) An illustration of the significantly lower progressive self-inhibition of dmPFC in the experimental as compared to the control group, as indicated by lower positive self-connectivity values given intrinsically negative self-connectivity parameters.

We applied a three-level hierarchical model to evaluate the progressive increase of resting-state effective connectivity during neurofeedback training using spDCM and Parametric Empirical Bayes (PEB) approaches^38–40^. For within-run spDCM, individual effective connectivity metrics were derived based on fitting the cross-spectra of the resting-state time-series within a fully-connected dmPFC and bilateral amygdala network architecture (Fig. 2A). Between-run PEB was used to model average and progressive (linear) effects across resting-state runs. Between-subject PEB was used to quantify the commonalities (i.e., the mean across both groups) and differences between the groups with respect to average and progressive effects across resting-state runs. We also quantified interactions between the main effect of group and covariates for individual neurofeedback learning slopes (linear trend over neurofeedback training runs) and valence differences (after vs. before training). Diagonal elements in the effective connectivity matrix A denote log-scaled self-connectivity in terms of the self-inhibitory prior, i.e. the self-inhibition, expressed as − 0.5·exp(A_i,i_). Positive self-connectivity values, indicate increased self-inhibition and estimated negative self-connectivity values indicate decreased self-inhibition (or disinhibition)^44,45^. Positive and negative connectivity parameters between network nodes indicate positive and negative directional influence (or interaction), respectively. Positive parameters in group differences indicate higher connectivity strengths in the experimental as compared to the control group, and vice versa for negative parameters.

For average connectivity across four resting-state runs, in both groups, we observed a significant disinhibition of all nodes (Fig. 2B; connectivity strength for dmPFC = − 0.23, Pp = 100%; for AMY_L_ = − 0.51, Pp = 100%; for AMY_R_ = − 0.32, Pp = 100%), as well as a negative top-down influence from dmPFC onto the bilateral amygdala (connectivity strengths = − 0.17, Pp-values = 100%). The average effect in the experimental as compared to the control group was associated with larger self-inhibition of dmPFC (connectivity strength = 0.10, Pp = 98%) and AMY_L_ (connectivity strength = 0.15, Pp = 100%), as well as lower self-inhibition of AMY_R_ (connectivity strength = − 0.08, Pp = 96%). The progressive effect across four resting-state runs in both groups was associated with a significant dmPFC self-inhibition in both groups (Fig. 2C; connectivity strength = 0.15, Pp = 99%), significant positive influence from dmPFC onto AMY_R_ (connectivity strength = 0.11, Pp = 97%) in both groups, and significantly lower dmPFC self-inhibition in the experimental group as compared to the control group (connectivity strength = − 0.13, Pp = 99%). The average (Fig. 2B) and progressive (Fig. 2C) effect in our PEB DCM model can be thought of as a line intercept and slope for all modeled parameters, respectively, which is visualized for the significant progressive modulation of the dmPFC self-inhibition (Fig. 2F). Thus, the progressive self-inhibition of dmPFC across four resting-state runs in the experimental group was significantly lower as compared to the control group, which is reflected in the lower slope for the experimental group.

In the interaction group × neurofeedback slope, for the average effect, we found a significant dmPFC self-inhibition (connectivity strength = 0.34, Pp = 96%) and AMY_R_ disinhibition (connectivity strength = − 0.47, Pp = 99%), which indicates that larger learning slope is associated with larger dmPFC self-inhibition and AMY_R_ disinhibition. In this interaction, for the progressive effect, we found a significant AMY_R_ self-inhibition (connectivity strength = 0.55, Pp = 99%), which indicates that larger learning slope is associated with larger progressive self-inhibition of AMY_R_. In the interaction group × valence difference, for the average effect, we found a significant dmPFC disinhibition (connectivity strength = − 0.29, Pp = 100%) and a negative AMY_L_ to AMY_R_ connectivity (connectivity strength = − 0.19, Pp = 98%). Surprisingly, in this interaction, for  the progressive effect, we found a significant AMY_R_ disinhibition (connectivity strength = − 0.29, Pp = 99%), which indicates that larger valence difference due to neurofeedback training is associated with larger progressive disinhibition of AMY_R_.

For both groups, we found that the average and progressive effects across four resting-state runs were best explained by the top-down connectivity model (Fig. 2D,E; Pp = 77% and Pp = 52%). The top-down model was also more evident in the experimental group as compared to the control group for average (Pp = 69%) and progressive (Pp = 53%) effects. We found that the between-subject covariates of neurofeedback learning slopes and valence differences interacted with the group effect, and the interaction was best explained by the top-down model (slope average effect Pp = 55% and progressive effect Pp = 51%; valence average effect Pp = 53% and progressive effect Pp = 53%).

### Resting-state data-driven spatiotemporal patterns by tICA

**Figure 3:**
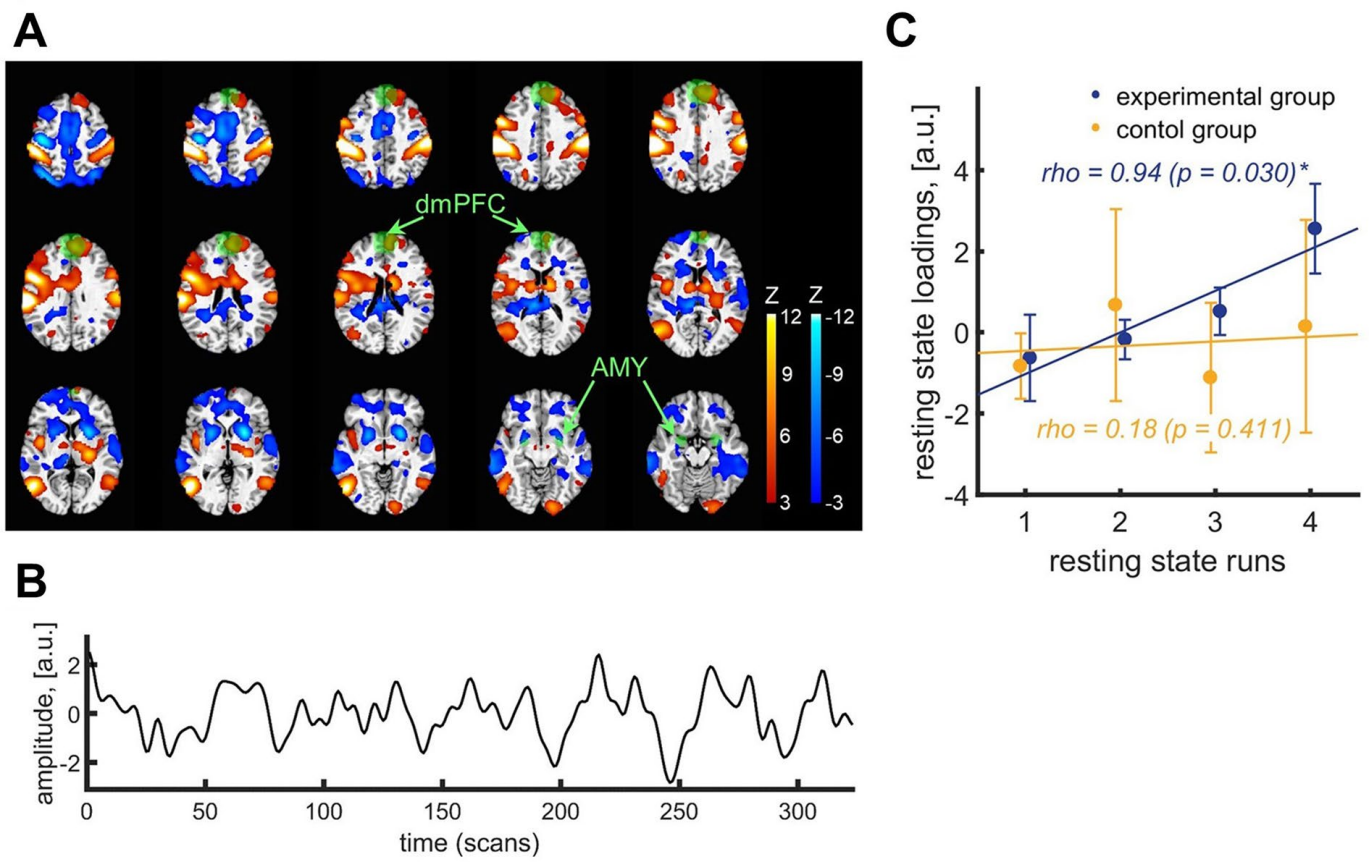
Spatiotemporal pattern associated with progressive increase of spontaneous resting-state activity across connectivity-based neurofeedback training. (**A**) Thresholded IC3 spatial map (decomposition #58; posterior probability, p > 0.5), the presence of which increased across four resting-state runs in the experimental but not in the control group, and (**B**) its associated time-series. Anatomical masks for dmPFC and bilateral amygdala ROIs that were used for neurofeedback were highly consistent with orthogonal positive (dmPFC) and negative (amygdala) subIC3s and indicated in green (Fig. 1). (**C**) Progressive increase of IC3 presence across four resting-state runs in the experimental but not in the control group (one-tailed Pearson correlation).

We performed data-driven tICA^41,42^ to investigate whether spatiotemporal patterns of spontaneous resting-state activity are modulated by learning positive-social emotion regulation effective connectivity neurofeedback. TICA explains observations using time × space × run domains, such that an independent component (IC) with a specific spatial map and time-series is weighted for each run. To assess the progressive modulation of ICs, we then computed a linear regression of each IC weights across four resting-state runs. For each decomposition in the dimensionality range from #50 to #80 ICs, in the experimental group we found a highly consistent single IC that showed a significant increase of presence across resting-state runs over neurofeedback training (two-tailed one-sample t-test on IC slopes, t-values > 5.35, p-values < 0.046, FDR-corrected for multiple comparisons across all 2015 ICs, q < 0.05; decompositions #64, 65 and 77 had the same IC which did not survive FDR correction). These ICs were highly spatially consistent across the performed decompositions (spatial cross-correlation given a rho > 0.25 threshold; rho-values > 0.61, p-values < 0.001). In addition, for post-hoc comparison, among these 29 ICs, 21 ICs had a significantly different increase of presence across resting-state runs in the experimental compared to the control group (two-tailed two-sample t-test between slopes, t > 2.21, p < 0.046 unc). To report key findings, we selected IC3 of the decomposition #58 (83.3% of total variance) with maximum 7.9% of total variance across 21 ICs (Fig. 3).

We observed the progressive increase of the IC3 presence across four resting-state runs over neurofeedback training in the experimental group (one-tailed Pearson rho = 0.94, p = 0.030) but not in the control group (one-tailed Pearson rho = 0.18, p = 0.411) (Fig. 3C). IC3 consisted of two anticorrelated subcomponents (Table 1, Fig. 3A). Positive subIC3 included prefrontal, premotor and occipital cortices as well as bilateral insula. Negative subIC3 included anterior cingulate cortex (ACC), motor areas, temporal and occipital cortices as well as bilateral thalamus, putamen, amygdala, and hippocampus. We confirmed that anticorrelated subICs were consistent with anatomical ROI masks within which most active voxels were dynamically selected to estimate the connectivity-based feedback signal (Fig. 3A)^29^. Positive subIC3 showed a spatial overlap with dmPFC (66% voxels), while negative subIC3 had an overlap with bilateral amygdala (AMY_L_: 57%, AMY_R_: 63% voxels) anatomical ROI masks. Note that decomposition #58 was consistent with conventional template resting-state networks (RSNs)^46^, as shown by cross-correlation between positive and negative subIC3s and RSN templates (Table 2; spatial cross-correlation as implemented in FSL given a rho > 0.25 threshold^47^). Notably, IC3 showed significant spatial overlap with the visuospatial RSN (rho = 0.31).

**Table 1:** Brain areas within subICs of IC3 that increased presence across resting-state runs.

subIC	Anatomical cluster	Main peak MNI coordinate (x, y, z)			z-value
Positive	SFG	20/− 13	52/52	28/28	6.13/3.39
	dmPFC	5	52	28	8.42
	Premotor cortex	56/− 52	10/7	34/31	9.77/15.65
	SI	59	− 20	43	15.31
	SPL	− 25	− 53	73	8.39
	Supramarginal gyrus	− 61	− 26	43	22.08
	Lateral occipital cortex	53/− 52	− 56/− 65	1/− 2	8.58/15.22
	Posterior insula	38/− 43	− 2/− 2	16/7	5.68/9.62
	Caudate	11/− 16	1/− 5	19/16	10.29/8.04
	Primary visual cortex	14	− 92	− 8	8.65
Negative	Hippocampus/amygdala	32/− 22	− 17/− 11	− 20/− 26	− 5.84/− 5.40
	MFG	− 31	16	52	− 5.70
	vmPFC/rACC	11/− 4	49/49	1/1	− 5.70/− 6.51
	OFC	32/− 28	37/31	− 17/− 14	− 4.79/− 5.35
	SMA	5/− 10	− 5/− 5	55/52	− 7.42/− 8.72
	Primary motor cortex	23/− 40	− 26/− 17	64/52	− 8.31/− 11.97
	SII	8/− 7	− 38/− 35	58/58	− 5.31/− 5.78
	Precuneus	2/− 1	− 65/− 71	61/55	− 6.31/− 5.64
	Posterior parietal sulcus	32/− 40	− 65/− 65	58/58	− 8.46/− 10.32
	MOG	29	− 80	25	− 3.75
	ITG/MTG	62/− 67	− 41/− 38	− 11/− 8	− 6.46/− 8.06
	Temporal pole	41	10	− 26	− 4.42
	Thalamus	8/− 10	− 26/− 29	19/13	− 7.32/− 9.39
	Putamen	23/− 22	10/10	1/− 2	− 11.91/− 9.60

Main peak Montreal Neurological Institute (MNI) coordinates and statistics for brain areas within IC3 (#58 decomposition; posterior probability p > 0.5). Spatially anticorrelated subcomponents (subICs) of IC3 were labeled as positive and negative. Anatomical labels were validated using Neurosynth.org and the SPM Anatomy Toolbox^48^.

*SFG* superior frontal gyrus, *dmPFC* dorsomedial prefrontal cortex, *SI* primary somatosensory cortex, *SPL* superior parietal lobule, *MFG* middle frontal gyrus, *OFC* orbitofrontal cortex, *vmPFC* ventromedial prefrontal cortex, *rACC* rostral anterior cingulate cortex, *SMA* supplementary motor area, *SII* secondary somatosensory cortex, *MOG* middle occipital gyrus, *ITG* inferior temporal gyrus, *MTG* middle temporal gyrus.

**Table 2:** Spatial correlation of ICs of decomposition #58 with resting-state networks.

subIC	RSN	IC	Pearson rho
Positive	VSN	3	0.31
	Anterior SN	38	0.26
	Dorsal DMN	41	0.48
	Ventral DMN	25	0.36
	Right ECN	39	0.32
	Left ECN	24	0.39
	AN	54	0.43
	BGN	42	0.39
	PN	47	0.43
	LN	51	0.25
	SMN	50	0.29
	Primary VN	34	0.47
	High VN	4	0.56
Negative	High VN	34	0.25
	Posterior SN	26	0.30

Pearson rho values above 0.25 were considered significant^47^. The ‘high VN’ correlated with positive subIC4, and to a lesser extent with negative subIC34.

*VSN* visuospatial network, *SN* salience network, *DMN* default mode network, *ECN* executive control network, *AN* auditory network, *BGN* basal ganglia network, *PN* precuneus network, *LN* language network, *SMN* sensorimotor network, *VN* visual network.

For the control group, we did not observe any resting-state ICs that significantly increased across resting-state runs (two-tailed one-sample t-test on slopes, t-values < 0.27, p-values > 0.9, FDR-corrected for multiple comparisons across all 2015 ICs, q < 0.05).

### Resting-state functional connectomics

**Figure 4:**
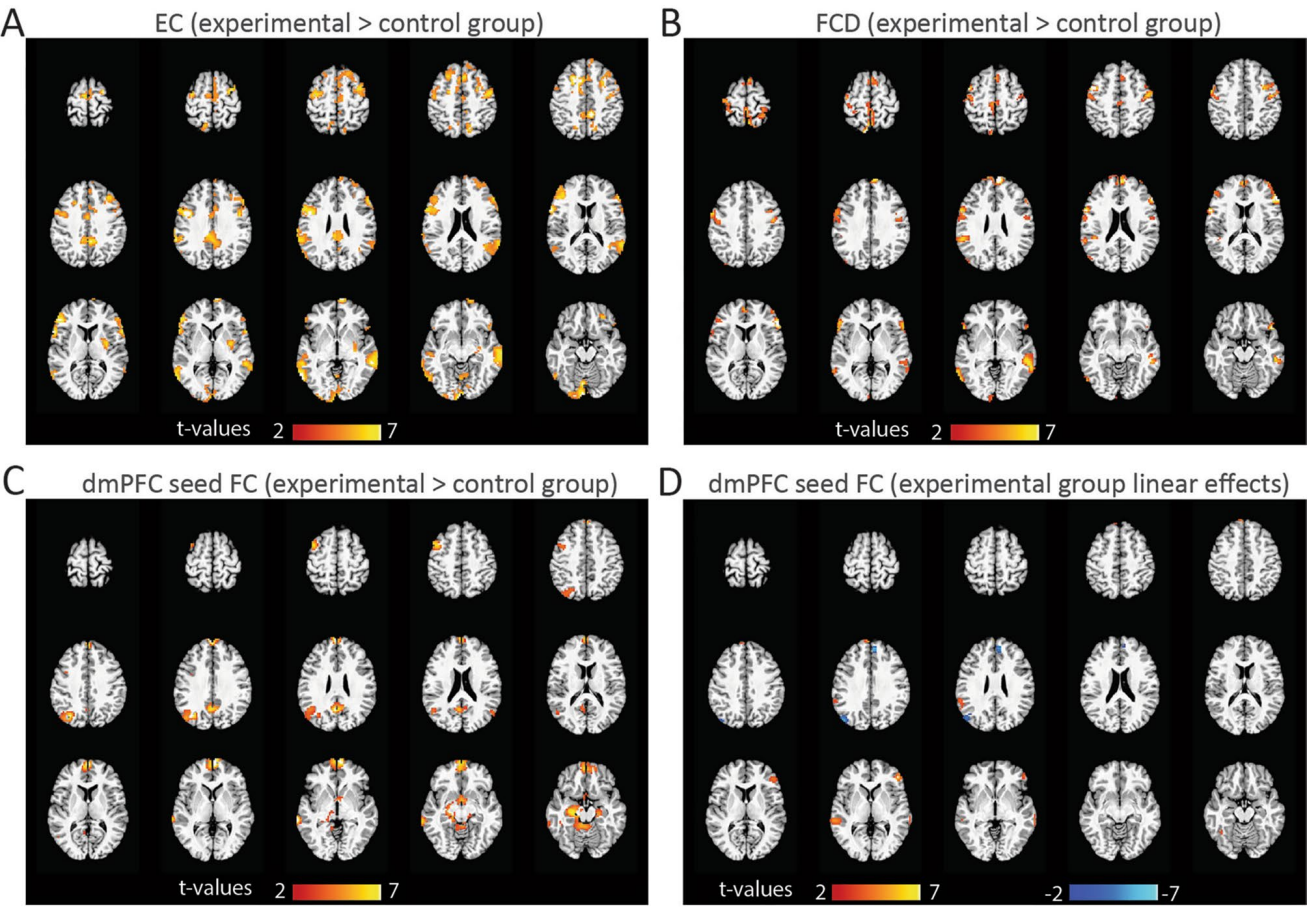
Functional connectomics. (**A**,**B**) Resting-state eigenvector centrality (EC) and functional connectivity density (FCD) were larger in the experimental group (compared to the control group) in, among other regions, the dmPFC, OFC, vmPFC, IFG, vlPFC, SPL, MTG and ITG (Table 3; peak-level FWE correction, p < 0.05). (**C**) Significantly larger resting-state dmPFC seed-based FC in the experimental group in, among other regions, the dmPFC, hippocampus, vmPFC, MTG, PCC and IPL. (**D**) Significantly larger progressive effects of neurofeedback training across resting-state runs (linear contrasts implemented in SPM12 for second-level flexible ANOVA; red map, positive effect in SFG/dmPFC and vlPFC; blue map, negative effect in dmPFC and IPL). For illustration purposes, maps were thresholded at p < 5e−5 unc. (**A**) and at p < 0.001 unc. (**B**–**D**).

We used eigenvector centrality (EC) mapping to reveal resting-state modulations of brain areas that are central within the small‐world network and strongly correlated with other nodes^43^. For whole-brain EC estimates, we found an average increase of the EC across resting-state runs in the experimental as compared to the control group in several brain areas, including dmPFC, IFG, vlPFC, SPL, ITG, and MTG (Table 3, Fig. 4A). We used conventional whole-brain functional connectivity density (FCD) analysis to identify resting-state modulations of brain areas with large long-range correlations. For FCD estimates, we found a very similar to EC average increase of FCD across resting-state runs in the experimental as compared to the control group in the TICA brain areas listed above, IPL and PG (Table 3, Fig. 4B). For EC and FCD estimates, we did not find the significant interaction group × run, as well as progressive modulations across resting-state runs in the experimental or control group.

**Table 3:** Resting-state functional connectomics.

Connectivity	Anatomical cluster	Main peak MNI coordinate (x, y, z)			t-value
EC (experimental > control average)	dmPFC	6	66	26	5.37
	OFC	30	39	− 19	6.47
	vmPFC	9	72	− 1	8.60
	dlPFC	36	33	38	6.18
	Hippocampus	− 21	− 15	− 16	5.39
	vlPFC (BA45)	57/− 45	30/33	20/11	6.70/7.13
	Insula	33	− 6	5	6.61
	IFG (BA44)	60/− 60	15/15	5/8	6.97/8.01
	MTG	60/− 60	− 39/− 39	− 4/− 4	7.32/6.30
	SPL	− 12	− 63	59	6.16
	MCC	0	− 3	35	6.60
	PCC	− 3	− 39	29	6.36
	Posterior ITG/MTG	− 60	− 66	− 4	7.55
	Putamen	33	− 6	5	6.61
	SMA	6	24	50	6.41
	Primary motor cortex	42/− 36	− 9/− 6	47/59	6.28/7.23
	Primary visual cortex	− 3	− 93	− 16	8.16
FCD (experimental > control average)	dmPFC	6	63	26	9.11
	IFG (BA44)	− 57	9	14	7.97
	vlPFC (BA45)	57	30	14	11.37
	SPL	− 3	− 60	65	7.28
	Posterior ITG/MTG	− 57	− 69	− 7	6.15
	IPL (area PF)	− 57	− 42	26	6.17
	Primary motor cortex	51/− 57	− 6/0	47/44	6.32/6.61
	MTG	60/− 66	− 39/− 54	− 4/− 1	6.70/6.45
	PG	− 48	6	50	8.61
dmPFC FC (experimental > control average)	dmPFC	0	57	32	8.67
	Hippocampus	− 27	− 18	− 19	7.12
	vmPFC	3/− 3	57/57	− 13/− 13	6.0/6.50
	PCC	− 3	− 60	26	7.10
	MTG	− 69	− 33	− 1	8.32
	IPS/IPL (posterior, PG)	− 30	− 70	35	7.46
	MFG	− 45	12	53	6.80
	BF	3	0	− 7	5.64
dmPFC FC (experimental positive linear)	SFG/dmPFC	− 3	60	35	6.37
	vlPFC (BA45)	48	39	2	5.46
dmPFC FC (experimental negative linear)	dmPFC	6	48	32	5.93
	IPL (posterior, PG)	− 48	− 75	32	5.44

Main peak Montreal Neurological Institute (MNI) coordinates and statistics (peak-level FWE correction, p < 0.05).

*EC* eigenvector centrality, *FC* functional connectivity, *FCD* FC density, *dlPFC* dorsolateral prefrontal cortex, *vlPFC* ventrolateral prefrontal cortex, *IFG* inferior frontal gyrus, *MCC* middle cingulate cortex, *PCC* posterior cingulate cortex, *IPL* inferior parietal lobule, *IPS* inferior parietal sulcus, *BF* basal forebrain.

We used conventional seed-based FC analysis to investigate modulations of resting-state dmPFC connectivity with the rest of the brain. We found larger average increase of FC across resting-state runs in the experimental as compared to the control group in dmPFC, hippocampus, vmPFC, IPS/IPL, MTG, PCC, MFG, and BF (Table 3, Fig. 4C). We did not find the significant interaction group × run. However, in the experimental group, the progressive modulation of dmPFC FC across four resting-state runs was associated with a significant increase of its self-connectivity with the SFG/dmPFC cluster and its connectivity with the vlPFC. In addition, the progressive modulation of dmPFC seed-based FC was associated with a significant decrease of its self-connectivity with another part of dmPFC cluster and its connectivity with IPL (Table 3, Fig. 4D). Note that self-connectivity clusters did not overlap and may highlight a heterogenous functional sub-specialization between SFG and dmPFC areas in positive-social emotion regulation^30,35^. We did not observe progressive FC modulations in the control group.

To directly link learning effects and resting-state EC, FCD and seed-based FC estimates, we identified brain areas associated with an interaction between the neurofeedback slope and valence difference covariates and the corresponding main effects of group (Supplementary Tables S1 and S2). Brain areas identified with these interactions were substantially different from those identified for main effects of group albeit within a consistent set of areas engaged in neurofeedback of positive-social emotion regulation.

## Discussion

Here we show that during successful effective connectivity neurofeedback training of positive-social emotion regulation in healthy participants, there is convergent evidence for progressive reorganization of effective connectivity, functional connectivity, and spatiotemporal patterns of activity during resting-states at the end of each neurofeedback session. These findings of progressive resting-state connectivity modulations extend previous real-time fMRI neurofeedback work that primarily focused on changes of functional connectivity after as compared to before training.

Our task-related neurofeedback findings demonstrate that learning positive-social emotion regulation is associated with an increase of direct influence of dmPFC onto bilateral amygdala^29^. This is consistent with studies demonstrating that emotion regulation is associated with increased top-down signals that stem from prefrontal cortices which modulate bottom-up emotional responses in amygdala^15,28–30,49–52^ and further confirms brain networks involved in the upregulation of positive versus negative emotions have common components^24,30,35,53–58^.

Our resting-state effective connectivity analysis revealed significant average and progressive (linear trend over resting-state runs) effects associated with learning effective connectivity-based neurofeedback related to positive-social emotion regulation (Fig. 2). Most importantly, progressive self-inhibition of dmPFC across four resting-state runs was significantly lower in the experimental group as compared to the control group and was found to modulate self-inhibition processes in bilateral amygdala. These findings are in a good agreement with an increase of dmPFC activity during the neurofeedback task, as well as a decrease in AMY_R_ activity and an increase of top-down dmPFC to amygdala connectivity in the experimental group (reported in our previous publication; Fig. 1)^29^. The fact that the control group also demonstrated a significant increase in dmPFC self-inhibition accords well with the significant increase of AMY_L_ activity during neurofeedback runs in the control group^29^, indicating a regulatory mechanism that is opposite to that found in the experimental group. The dmPFC is thought to exert influence onto other frontal and limbic regions involved in emotion processing, such as vmPFC and amygdala, to which dmPFC and vmPFC are both densely connected^50,52^. We suggest that progressively lower self-inhibition of the dmPFC could modulate processes in bilateral amygdala, which extends the regulatory role of dmPFC to neurofeedback based on positive-social emotion regulation^30^.

Neurofeedback learning slopes and valence differences also demonstrated associations with dmPFC and AMY_R_ self-inhibitions. We found that larger learning slopes were associated with larger average dmPFC self-inhibition and average AMY_R_ disinhibition, which is characteristic of progressive dmPFC self-inhibition in the experimental group (Fig. 2F) and the fact that training-induced activity in dmPFC increases and in AMY_R_ decreases across neurofeedback runs^29^. Our findings also highlight that positive mood induction is related to average disinhibition of the dmPFC and progressive disinhibition of AMY_R_. Conversely, larger progressive self-inhibition of AMY_R_ is associated with larger learning slope, and larger progressive disinhibition of AMY_R_ is associated with larger valence difference. These findings are surprising regarding the flip of the dmPFC and AMY_R_ self-connectivity parameter in the main effect of group interactions with learning slopes and valence differences. However, due to this flip, they do not contradict that individual learning slopes and valence differences correlate positively in both groups^29^. We suggest that these flips could be due to the specific group differences in DCM parameters. Alternatively, these findings could indicate that although experimental group learned the top-down emotion regulation mechanism, concurrent positive mood induction remains characteristic of the progressive dmPFC self-inhibition in both groups albeit it is lower in the experimental group. We speculate that training a top-down emotion regulation mechanism could therefore indicate a more balanced positive mood induction as compared to training just upregulation of positive emotions, which could be justified in future research. Comparing different model architectures, we confirmed that the top-down model explained average and progressive effects remarkably better than the alternative models and that it was more evident in the experimental as compared to the control group (Fig. 2D,E). These findings are highly consistent with top-down task-related neurofeedback learning effects related to the current paradigm^29^.

To complement the hypothesis-driven model-based effective connectivity analysis, we used the data-driven exploratory analysis to investigate progressive modulations of whole-brain spontaneous spatiotemporal activity patterns (Fig. 3). Highly consistent progressive modulations were observed in the experimental but not in the control group. Progressively modulated IC comprised of two anticorrelated subcomponents (subICs), both encompassing brain regions involved in emotion regulation^51^, neurofeedback learning^59^, salience^60,61^, memory^62^, and sensory processing^63^. More specifically, positive subIC involved parts of the emotion regulation circuit^50,64^, including right dmPFC, SFG, insula and caudate nucleus, as well as primary visual, somatosensory cortices, SPL, supramarginal gyrus, lateral occipital and premotor cortices that could be engaged in visuospatial processing^63^. Negative subIC, among other regions, included hippocampus/amygdala, MFG, vmPFC/rACC, OFC, SMA, putamen, and MOG which could be engaged in memory processes^62^, emotion regulation and discrimination^51,65^. For instance, vmPFC is typically engaged in affective evaluations^50^, and during self-referential positive-social emotion regulation the vmPFC function could be governed by regulatory inputs originated in dmPFC and SFG^30^. Moreover, identified in positive subIC, the insula is commonly implicated in salience processing^60,61^, neurofeedback learning^59^ and emotion regulation^50^; in the current study, this may represent neurofeedback associated salience monitoring, autonomic regulation, interoceptive processing, and bodily representations of affect during emotion regulation^66^. Primary and secondary sensory areas could be engaged in the generation of emotional responses as suggested by the theory of constructed emotion^67^. This theory implies that the brain does not simply react to incoming sensory inputs, but rather anticipates these inputs based on past experiences in a predictive manner^68^. Incoming sensations are constantly compared to predictions, generated based on previous experience, forming a prediction error. When a prediction error is sufficiently large, the internal model that stores information about past experiences is updated, which results in more accurate predictions for similar future situations. In light of this theory, predictions initiated in the dmPFC, vmPFC, vlPFC, ACC, MCC, anterior insula, SMA and premotor cortex could be transmitted to the hypothalamus and brainstem to regulate autonomic, neuroendocrine and immune systems (visceromotor predictions); to the motor cortex (motor predictions); as well as to sensory cortices (sensory predictions), including somatosensory and primary visual cortices^67,69^. Identified brain regions were also consistent with those typically engaged in neurofeedback, e.g. vlPFC, dlPFC, ACC, premotor, temporo-parietal and occipital cortices, anterior insula, putamen, caudate and thalamus^59^.

The findings of ROI network model-based effective connectivity and whole-brain data-driven spatiotemporal activity insights are consistent with broadly used functional connectivity analyses based on voxel-wise Pearson correlations. The average increase of rs-FC across runs was observed in multiple areas in the experimental group as compared to the control group, most notably in the dmPFC, hippocampus, vmPFC, IFG (BA44), vlPFC (BA45), SPL, MTG and MFG, in terms of the EC and FCD estimates (Fig. 4). In the experimental group dmPFC seed-based FC analysis, we observed the progressive increase of its connectivity with SFG/dmPFC and vlPFC (BA45) and the progressive decrease of its connectivity with another part of dmPFC and IPL (posterior PG). The progressive increase of dmPFC and vlPFC connectivity could indicate an improvement in positive-social emotion regulation during neurofeedback training, which accords well with a concurrent increase of activity in dmPFC and vlPFC, indicating a regulatory success during reappraisal^70^. In addition, vlPFC could be implicated in emotion regulation processes by selecting or inhibiting goal-appropriate responses and interpretations to emotional situations^52^ as well as in diminishing self-reports of negative affect via impacting amygdala reactivity^50^. SPL and MFG are considered a part of the dorsal attention network, and areas including IPL are a part of the ventral attention network, critically implicated in visuospatial function^71^. Increased prefrontal and limbic connectivity along with increased connectivity within visuospatial RSNs, as also indicated by tICA, may indicate increased integration of visuospatial information in the scene during positive-social imagery. Decrease of connectivity between dmPFC and IPL (posterior PG) could be associated with strong referencing to one’s self during the control of one’s own emotions^30^ and related to visuospatial processing by shifting focus towards distant space^63^. In addition, the posterior PG has been shown to be involved in shifting the attention system toward stimuli that have high salience^72^. Thus, a progressive decrease of dmPFC-IPL connectivity could indicate a counter-mechanism against this shift of visuospatial and attention systems during learning self-referential emotion regulation and top-down dmPFC control over bilateral amygdala.

The hippocampus is one of the most prominent brain structures related to memory encoding, processing and learning. It is implicated in the cognitive control of emotions, supporting the engagement of prefrontal, cingulate and limbic cortices in the evaluation and regulation of emotions^50,62^. Consistent with this view, we observed an average increase in both hippocampal connectivity and connectivity between hippocampus and dmPFC (Table 3). We also observed that successful neurofeedback learning (learning slope) was associated with an increase of average dlPFC connectivity, thalamus connectivity, and connectivity between dlPFC and dmPFC (Supplementary Table S1). Since dlPFC is known for maintaining information in working memory^62^, and thalamus is known for integrating information processing across multiple functional networks^73^ and engaging in neurofeedback-related learning^74^, consistent increases in resting-state functional connectivity of hippocampus, thalamus and dlPFC could indicate an engagement of memory and learning processes during neurofeedback training. Another consistent observation is related to the increase of vmPFC connectivity and connectivity between vmPFC and dmPFC engaged in affective evaluation^50^ and positive-social emotion regulation processes^30^, as well as of OFC, rACC, and IFG connectivity engaged in cognitive control of memory representations^62^. Notably, dmPFC and SFG appear in the subIC orthogonal to hippocampus/amygdala, vmPFC/rACC, and OFC, which conforms with the dominance of the trained top-down regulatory mechanism, upregulation of dmPFC, and downregulation of bilateral amygdala activity in task-related neurofeedback and transfer runs^29^. We also found that increased hippocampus, dlPFC, SFG, insula and thalamus connectivity was associated with larger valence differences (Supplementary Table S2), linking observed memory and learning processes with positive mood induction.

Although the applied data sample size is rather small, the current dataset is highly consistent and generates distinct significant findings between experimental and control groups in terms of behavioral estimates, neurofeedback task-related brain activity and connectivity estimates, as well as complementary model-based and data-driven estimates used to assess resting-state connectivity. This is likely due to the large amount of low-variability data collected from each participant, including 3-days of neurofeedback training with six relatively long training runs and a relatively narrow suggested positive-social emotion regulation strategy (Supplementary Table S3), which resulted in consistent training across participants. Despite high similarity and consistency across EC, FCD and seed-based FC findings, there are differences which could be attributed to intrinsically different aspects of studied resting-state connectivity features, different sensitivity to resting-state fluctuations, as well as differences between the individual learning slopes and valence differences covariates.

To summarize, positive-social emotion regulation effective connectivity neurofeedback was characterized with specific learning effects, which included (i) increased valence ratings, (ii) increased top-down regulation mechanisms exerted from dmPFC onto bilateral amygdala during training, transfer runs and resting-states at the end of training sessions, (iii) increased dmPFC activity during training and transfer runs, (iv) decreased right amygdala activity during training, (v) lower dmPFC self-inhibition during resting-states at the end of training sessions in the experimental as compared to the control group, (vi) increased resting-state functional connectomics in emotion regulation, neurofeedback learning, visuospatial and memory networks, and (vii) linked behavioral, task-related and resting-state alterations. We suggest that lower progressive dmPFC self-inhibition in the experimental group could be associated with its increased connectivity with other regions, as evidenced by progressively increased patterns of spatiotemporal activity and functional connectomics. Our findings highlight a large-scale synergy between neurofeedback task-related and resting-state connectivity during intense neurofeedback training and moves towards better understanding of neurofeedback learning mechanisms.

Between-day restoration and consolidation effects related to rest and sleep take place within non-neurofeedback studies, for example in studies focusing on memory processing^75–77^, imagery learning^78^, as well as cognition and emotion regulation^79^. Since neurofeedback training typically requires multiple training runs spanned over daily training sessions with intermediate and between-day rest and sleep periods, it could be assumed that specific restoration and consolidation processes might take place between the neurofeedback training sessions. It was also suggested that larger increases in the neurofeedback signal across training sessions on different days than within daily training sessions could indicate a between-day consolidation effect^80^. Further research is needed to investigate whether resting-states at the end of neurofeedback training sessions could reflect the beginning of specific restoration and consolidation effects. Investigation of rest and sleep processes between neurofeedback training periods could be used to assess the efficacy of intermediate rests, and to help in optimizing the number of training sessions and rest days required for effective neurofeedback-based therapy.

## Methods

### Participants

Fifteen healthy volunteers from the local student/staff community (7 male, 8 female, age 26 ± 1 years) with normal or corrected-to-normal vision and no prior history of neurological or psychiatric diseases performed a 3-day neurofeedback training experiment^29^. The present study data sample is based on previously unpublished resting-state data from the published positive-social emotion regulation neurofeedback study^29^. The sample size of this preliminary study was selected based on the practical considerations, such as availability of scanner time and funding. Healthy participants were assigned sequentially to experimental (4 males, 5 females, 26 ± 5 years) and control (3 males, 3 females, 25.7 ± 2.9 years) groups, which allowed us to derive control (sham) feedback values based on values of the best performing participants in the experimental group. Experimental participants successfully acquired the positive-social emotion regulation through neurofeedback as compared to control participants^29^. Participants gave written informed consent to participate in the experiment, all methods used in this study were performed in accordance with the relevant guidelines and regulations of the University Hospital of Geneva, all experimental procedures used in this study were approved by the Ethics Committee of the University Hospital of Geneva. At the beginning of the experiment, participants were provided with written instructions describing experimental procedures, which stated that they would be asked to control the emotion regulation networks using induction of positive-social emotions. Behavioral testing included psychological questionnaires before the experiment and rating positive-social stimuli for valence and arousal before and after the experiment using the self-assessment manikin (SAM) scale (for details on experimental design and analyses, see the Supplementary Information).

### Resting-state fMRI runs

To test whether learning positive-social emotion regulation using DCM connectivity-based neurofeedback led to specific changes in resting-state spontaneous activity, conventional eyes-closed resting-state fMRI runs (6.1 min run duration) were acquired prior to neurofeedback training and after each training session (Fig. 1A). During resting-state runs participants were instructed to let their minds wander and avoid specific thinking and falling asleep, which was verified during debriefing. Whole-brain fMRI scans for resting-state runs were acquired with a multi-band gradient-echo T_2_*-weighted EPI sequence (333 scans, TR = 1100 ms, TE = 30 ms, 45 slices with 25% distance factor, 120 × 120 matrix, 1.8 mm^3^ voxels, flip angle α = 70°, bandwidth = 1.49 kHz/pixel, TE = 30 ms, multi-band acceleration factor = 3, GRAPPA with iPAT = 3) (for details on MRI data acquisition, see the Supplementary Information).

### fMRI data preprocessing

Resting-state fMRI data were preprocessed using SPM12 (FIL, Wellcome Trust Centre for Human Neuroimaging, UCL, London, UK) and in-house MATLAB scripts. The first 10 volumes were discarded to account for T1 saturation effects. The fMRI data preprocessing included realignment to the mean scan of each fMRI run, coregistration to the standard MNI structural template using DARTEL^81^, correction for geometric distortions^82–84^ and smoothing with 8 mm full-width-at-half-maximum (FWHM). Isotropic 1.8 mm^3^ fMRI scans were downsampled to isotropic 3 mm^3^ images to facilitate the computationally intensive eigenvector centrality mapping while keeping data preprocessing identical for all applied analyses. Physiological noise of fMRI runs was reduced by regressing out nuisance signals voxel-wise and band-pass filtering in the 0.008–0.15 Hz range. We regressed out the average and first principal component of white matter (WM) and cerebrospinal fluid (CSF) signals, the first-order polynomial trend, as well as 6 head motion parameters, derivatives for head motion parameters and scans with excessive motion (i.e., with framewise displacement > 0.9). Framewise displacement across scans was estimated as the combined difference of head motion displacements between subsequent scans^85^. The moderate low-pass frequency was set to 0.15 Hz, because of preceding neurofeedback training with relatively short regulation and baseline blocks (12 s). The preprocessing and analysis details associated with the neurofeedback training and transfer runs have been published before^29^.

### Neurofeedback task-related and resting-state effective connectivity by DCM

To provide the effective connectivity-based neurofeedback, two DCM model alternatives were compared in real-time, namely, top-down network model (from dmPFC onto bilateral amygdala) versus a bottom-up one (from bilateral amygdala onto dmPFC) using task-related bilinear DCM and Bayesian model comparison^29^. Such a comparison was expressed in terms of the logarithmic Bayes factor and allows to test which model architecture explains the acquired real-time time-series best, i.e., dominates. In our previous publication the experimental and sham groups were found to be significantly different in terms of the applied neurofeedback task-related effective connectivity estimates and concomitant learning effects.

Here, to investigate whether learning positive-social emotion regulation using task-related effective connectivity-based neurofeedback leads to progressive increase in resting-state effective connectivity within the trained network, we applied rest-related effective connectivity metrics based on three-level hierarchical model. We characterized within-run, between-run and between-subject effective connectivity using spectral DCM (spDCM) and Parametric Empirical Bayes (PEB)^44,86,87^ approach, as implemented in SPM12^38–40^. In contrast to task-related DCM, resting-state spDCM is developed to analyze effective connectivity during rest and based on fitting the cross-spectra of the observed time-series. To extract time-series for spDCM, we used individual static dmPFC and bilateral amygdala regions of interest (ROIs) defined as the disjunction of consistent dynamic ROIs in the last 3 neurofeedback training runs for each participant of the experimental group and first 3 neurofeedback runs for each participant of the control group. Specifically, for DCM-based neurofeedback training, most active voxels within preselected anatomical ROIs were identified dynamically using incrementally estimated general linear model (GLM) and updated across neurofeedback trials. The size of dynamic dmPFC ROIs did not differ across neurofeedback runs (for details, see Ref.^29^).

At the first-level, to infer the effective connectivity that best explains time-series for resting-state runs per participant, we estimated fully-connected DCM models consisting of three brain regions, namely: dmPFC and bilateral amygdala static ROIs (Fig. 2A). Because resting-state DCM does not imply modulatory and external stimuli, the model was specified based on endogenous connections (matrix A; 9 DCM parameters per run). PEB parameters are estimated using Bayesian model comparison (BMC) and averaging (BMA) over all possible reduced (nested) PEB model alternatives generated from the fully connected model. This is accomplished by the Bayesian model reduction (BMR) approach, i.e. switching on or off different combinations of connectivity parameters to generate large model space of reduced models for testing. The posterior probability (Pp) for each PEB parameter is estimated by comparing the evidence for all models which had the corresponding parameter switched on, versus all models which had that parameter switched off. At the second-level, using PEB, we estimated average (i.e., constant) and progressive (i.e., linear trend) connectivity parameters across four resting-state runs per participant (18 DCM parameters per participant). At the third level, to quantify the commonalities (i.e., the mean across both groups) and differences between the groups with respect to average and progressive effects across resting-state runs, second-level PEB parameters from each participant were submitted to a PEB of PEB model. We also modeled interactions between the main effect of group and covariates for individual neurofeedback learning slopes and valence differences. In addition, participants’ age and gender were modeled as covariates.

Finally, to estimate which model architecture best explains the commonalities and differences between experimental and control groups, we defined a set of three candidate (reduced) models, which varied in terms of their A-matrix connections i.e., fully-connected, top-down and bottom-up models (Fig. 2D). Connections between bilateral amygdala were excluded to be consistent with neurofeedback training DCM models (Fig. 1C). The model evidence for the winning model, its connection strengths and parameter posterior probabilities were derived using the BMC and PEB approaches^44,86,87^.

### Spatiotemporal patterns of spontaneous activity by tICA

DCM is a hypothesis-driven approach that requires prior knowledge about the target brain network dynamics and the modulatory influences. To investigate without prior assumptions whether learning positive-social emotion regulation via network model-based effective connectivity neurofeedback leads to a progressive increase of whole-brain spatiotemporal patterns of spontaneous resting-state activity, we performed data-driven tICA^41,42^. It was carried out within the MELODIC toolbox (Multivariate Exploratory Linear Decomposition into Independent Components, Version 3.15 package of FSL, fmrib.ox.ac.uk/fsl). Pooled resting-state runs of all participants were preprocessed, including masking of non-brain voxels, voxel-wise demeaning and normalization of the voxel-wise joint data variance. FSL whitens and reduces the dimensionality of the data using principal component analysis as an internal processing step before the ICA criterion is optimized. tICA explains observations using a tensor structure (time × space × run domains), such that a component with a specific spatial map and time-series is weighted for each run. Estimated IC maps were z-scored by dividing by the standard deviation of the residual noise and thresholded (posterior probability p > 0.5) by fitting a mixture model to the histogram of intensity values^41,42^.

The selection of the optimal number of ICs (i.e., model order/dimensionality, #IC) remains a challenge for techniques such as ICA and PCA^88,89^. It is known that higher model orders lead to more fragmented IC maps that might capture more local changes, while lower model orders are capturing more widespread effects. Thus, we checked the dimensionality range from #50 to #80 decompositions that explained between 78 and 95% of the total variance. Note that spatial–temporal ICs can encompass spatially anticorrelated subcomponents within a single time-series (subICs)^42^. The polarity of these components was synchronized across decompositions using cross-correlations across IC time-series as a reference. For consistent interpretation, anticorrelated spatial subICs were justified as positive (z > 0) and negative (z < 0) across all comparisons based on the cross-correlations between IC time-series.

For each IC and per decomposition, we computed a linear regression of IC weights (loadings) across four resting-state runs per participant using Pearson’s rho. To investigate within and between group effects, we used t-tests on Pearson rho values. Results were corrected for multiple comparisons using False Discovery Rate correction (FDR, q < 0.05) across 2015 comparisons (sum of ICs for 31 tICA decompositions).

For the decomposition that revealed IC with significant progressive changes across resting-state runs, we demonstrated the consistency of decomposed ICs with well-established resting-state networks (RSNs)^46^. Thus, positive and negative subIC spatial maps were compared to RSN templates using spatial cross-correlation with a threshold for Pearson rho > 0.25 (as implemented in FSL)^47^.

### Functional connectomics

To study the spatiotemporal structure of whole-brain functional components related to emotion regulation and neurofeedback learning networks, we performed conventional rs-FC analyses based on voxel-wise Pearson correlations. For each resting-state run, we estimated voxel-wise eigenvector centrality (EC), long-range FC density (FCD) and ROI seed-based FC maps. Whole-brain EC and FCD are methodologically different analyses with minimal parametrization, which were performed to study different aspects of functional connectomics. Voxel-wise EC analysis was performed to reveal modulations of brain areas that are central within the small‐world network and strongly correlated with other nodes^43^. EC mapping was applied to preprocessed whole-brain resting-state data using LIPSIA v3.1.0^43^ based on positive correlations without thresholding, similarly to previous studies^90–92^. Voxel-wise FCD was used to identify resting-state modulations of brain areas with large long-range correlations. Positive FCD maps were estimated voxel-wise as the number of Pearson correlations with the rest of the brain, which were above 0.7. For long-range FCD maps, local FCD maps (10 voxels extent threshold) were subtracted from total FCD maps.

Seed-based FC analysis was used to investigate modulations of resting-state dmPFC connectivity with the rest of the brain. As dmPFC FC seeds, we used individual static dmPFC ROIs as for resting-state effective connectivity analyses. To reduce calculations for the FC analysis, we limited them to grey-matter voxels. For dmPFC seeds (i.e., individual static dmPFC ROIs described above), we estimated whole-brain FC maps based on Pearson correlations without thresholding between the average seed time-series and the rest of the brain. Pearson correlations were Fisher-transformed.

For the whole-brain group-level analysis of FCD, FC and EC maps, we performed a three-way ANOVA with the fixed factors ‘run’ and ‘group’, and the random factor ‘subject’, as implemented in SPM12. Individual neurofeedback learning slope and valence difference, age and gender were modeled as covariates for all analyses, as well as size of dmPFC ROI covariate was additionally included for seed-based FS analysis. Statistical maps were corrected for multiple comparisons using whole-brain family-wise error correction (FWE, p < 0.05). Anatomical labels were validated using Neurosynth.org and the SPM Anatomy Toolbox^48^.

## Supplementary Information

Supplementary Information.
