# Supplementary material for: Progressive modulation of resting-state brain activity during neurofeedback of positive-social emotion regulation networks
Source: Sci Rep. 2021 Dec 3;11:23363. doi: 10.1038/s41598-021-02079-4 (PMC8642545; doi:10.1038/s41598-021-02079-4)
Supplement: Supplementary file 1 — Supplementary Information. [file 41598_2021_2079_MOESM1_ESM.pdf]

Supplemental Table

Table S1. Resting-state functional connectomics and neurofeedback learning slope.

connectivity	anatomical cluster	main peak MNI coordinate (x, y, z)			t-value
**EC (experimental > control)**	dmPFC	-9	66	20	5.12
	amygdala	-15	-15	-19	7.41
	OFC	-33	38	-13	6.09
	dlPFC	-36	36	41	6.71
	vlPFC (BA45)	-51	27	12	5.48
	posterior insula	51 / -48	-12 / -9	14 / 8	6.33 / 5.93
	MTG	69 / -54	-36 / -39	14 / -1	5.44 / 5.83
	ITG	27 / -36	12 / 6	-43 / -43	6.73 / 8.23
	SPL	-27	-57	44	6.29
	thalamus	12 / -6	-9 / -9	2 / 2	6.99 / 5.33
	occipital cortex	-45 / 48	-78 / -84	-4 / -4	5.36 / 5.66
	SMA	-6	3	62	5.38
	primary motor cortex	42 / -30	-3 / -3	47 / 47	6.41 / 6.48
	primary visual cortex	-3	-99	2	7.94
**FCD (experimental > control)**	dlPFC	-30	42	44	6.99
	dACC	9	27	14	6.29
	thalamus	12	-24	-1	7.39
	rACC/vmPFC	12	36	-7	6.87
	posterior insula	48 / -45	-18 / -12	8 / 11	5.91 / 7.29
	posterior ITG	-51	-57	-19	6.34
	IPS/IPL (area PF)	54 / -54	-39 / -42	56 / 56	8.08 / 6.79
	FFA	-39	-78	-19	7.02
	FFG	24 / -30	15 / 0	-43 / -49	7.48 / 8.31
**dmPFC FC (experimental > control)**	dlPFC	45 / -36	35 / 30	35 / 47	8.56 / 5.86
	vlPFC (BA45)	48 / -45	48 / 48	-1 / 2	7.00 / 5.86
	PFC	9 / -6	72 / 72	8 / 8	6.57 / 8.39
	OFC	-21	30	-13	6.58
	SFG/MFG	-24	3	53	5.76
	SPL	9 / -6	-75 / -78	56 / 53	7.36 / 6.93
	IPS/IPL (area PF)	51 / -39	-45 / -54	56 / 56	6.48 / 6.18

Main peak Montreal Neurological Institute (MNI) coordinates and statistics (peak-level FWE correction, $p < .05$). Anatomical labels were validated using Neurosynth.org and the SPM Anatomy Toolbox[1]. EC – eigenvector centrality, FC – functional connectivity, FCD – FC density. dmPFC – dorsomedial prefrontal cortex, OFC – orbitofrontal cortex, dlPFC – dorsolateral prefrontal cortex, vlPFC – ventrolateral prefrontal cortex, MTG – middle temporal gyrus, ITG – inferior temporal gyrus, SMA – supplementary motor area, vmPFC – ventromedial prefrontal cortex, rACC – rostral anterior cingulate cortex, dACC – dorsal ACC, IPL – inferior parietal lobule, IPS – inferior parietal sulcus, FFA – fusiform area, FFG – fusiform gyrus, MFG – middle frontal gyrus, SPL – superior parietal lobule.

Table S2. Resting-state functional connectomics and valence difference.

connectivity	anatomical cluster	main peak MNI coordinate (x, y, z)			t-value
**EC (experimental > control)**	hippocampus	-33	-21	-20	5.23
	thalamus	9	-18	5	6.54
**FCD (experimental > control)**	dlPFC	-27	42	44	6.31
	medial PFC	-9 / 12	33 / 33	41 / 38	7.11 / 6.04
	thalamus	12	-18	-4	6.29
	insula	42 / -36	-15 / 0	-10 / -7	5.34 / 5.23
	anterior insula	36 / -45	27 / 24	8 / 5	6.40 / 9.53
	posterior ITG/MTG	-51	-60	-16	6.09
	ITG	60 / -57	-18 / -12	-25 / -37	7.22 / 5.90
	MTG	33	18	-37	5.46
	IPS/IPL (posterior, PG)	-39	-62	32	7.55
	rACC/vmPFC	9	27	-10	6.63
	MFG	48 / -42	21 / 15	47 / 47	6.43 / 8.76
**dmPFC FC (experimental > control)**	SFG	15 / -15	39 / 39	53 / 53	6.51 / 6.98
	PFC	18 / -30	69 / 57	11 / -1	5.66 / 5.93
	MFG	39 / -45	24 / 21	53 / 47	9.87 / 8.02
	rACC/dmPFC	15	45	20	7.60
	ITG	-51	-6	-43	6.82
	IPS/IPL (area PF)	51 / -45	-54 / -54	50 / 56	7.40 / 5.38
	ITG	60 / -60	-39 / -39	-28 / -25	6.83 / 6.40

Main peak Montreal Neurological Institute (MNI) coordinates and statistics (peak-level FWE correction, $p < .05$).

Table S3. Reported **s**trategies that were used in the last neurofeedback training session.

Group	Participant	Rho	Strategy
**EG**	S1	.79	Focus on actual feelings related to the depicted situations.
	S2	.15	Comfort people shown in the images.
	S3	.45	Imagine funny memories related to the depicted situations.
	S4	.38	Imagine myself in joyful interaction with the depicted situations.
	S5	.27	Socializing with people in the depicted situations.
	S6	.81	Imagine to take part in the depicted positive situations.
	S7	-.20	Relaxing, imagine people related to the depicted situations.
	S8	.35	Imagine own involvement in the depicted situations; control emotions when they emerge.
	S9	.65	Imagine to talk to the people shown in the image.
**CG**	S1	.32	Associate colors with the depicted situation.
	S2	-.40	Imagine situations that are less emotional.
	S3	-.65	Create a personal, emotional connection to the people depicted in the images.
	S4	.21	Imagined myself interacting with the people shown in the pictures.
	S5	-.07	Imagine myself with friends, thinking of past experiences related to the depicted situations.
	S6	.32	Remember past experiences related to the depicted situations.

EG – experimental group; CG – control group; Rho – slope of the individual learning curves (Pearson rho).

Supplemental Results

Neurofeedback task-related learning effects

The analyses of the neurofeedback task-related learning effects have been published before[2]. There was no effect of unequal group sizes on observed differences between experimental and control group behavior. Specifically, the increase in valence ratings in the control group is not significant, assuming equal group sizes between the experimental and the control group and is thus not an effect of unequal sample size ($t_9 = \sqrt{9}/\sqrt{6} \cdot t_6$; $t_9 = 1.63$, $p_9 = 0.07$).

Follow up tests also indicated that arousal ratings and psychological questionnaire scores did not change with training. Participants in both groups showed indistinguishable self-reported attentional effort (two-tailed two-sample t-test, $t(13) = 0.32$, $p = 0.76$; rating experimental group: 4.33±0.87, rating control group: 4.17±1.17), yet the training success diverged substantially (**Fig. 1E,F**). Feedback reward levels in both groups were also identical, and participants in the control group were unaware that they had received sham feedback. Thus, attentional and motivational factors cannot explain the reported neurofeedback findings. In debriefing, both groups consistently reported similar neurofeedback strategies related to personal engagement into the depicted positive social situations (**Table S3**). Although with somewhat greater variability, participants in the control group used similar strategies, but they nevertheless failed to learn self-regulation. This indicates that more implicit learning mechanisms attributable to operant conditioning based on reinforcement by the feedback might be a factor[2,3].

Supplemental Methods

Neurofeedback training experimental design and analyses

Our study employed a 3-day neurofeedback study design with an experimental and control (sham) group. Specifically, it included 3 neurofeedback training sessions spread over 4.7±0.8 days, pre-/post-training behavioral tests and questionnaires, transfer runs and resting-states (**Fig. 1A**). For neurofeedback training, transfer and behavioral test runs, stimuli consisted of balanced and randomized sets of positive-social photographs (684 images; 504 images for neurofeedback training with normative valence 6.73±0.92, arousal 4.48±1.00; 180 images for pre-/post- training tests with normative valence 6.40±0.93, arousal 4.22±0.92) and neutral non-social (objects) photographs (696 images; 630 images for neurofeedback training with normative valence 5.34±0.72, arousal 3.67±0.97; 66 images for pre-/post-training tests with normative valence 5.41±0.52, arousal 3.65±0.84) taken from the International Affective Picture Set[4], Nencki Affective Picture System[5], and Geneva Affective Picture Database[6]. Images were presented in pseudo-randomized order, once to each participant. We used slightly less emotional images during testing to avoid ceiling effects and to thereby allow detection of training-related changes. We also wanted to test if learned self-regulation transfers not only to situations without neurofeedback, but also to situations with different valence levels. For additional details on stimuli, refer to the original publication[2].

Every training session consisted of two training runs that included 7 neurofeedback trials each (17.5min run duration). Each neurofeedback trial was composed of interleaved four regulation and five baseline blocks of 12s duration (**Fig. 1B**). For each block, 3 images spanning 12° visual angle were presented to participants. During regulation blocks, moderately positive-social images were presented, and participants were asked to feel-positive and induce their positive-social emotions to maximize the feedback signal, for example, by balancing emotional and social components of their imagery while trying to actively immerse themselves into the presented situation. We were not prescriptive about the exact strategy that participants should adopt during learning within the suggested direction of positive-social emotion regulation[2]. During baseline blocks, participants were instructed to passively observe images of neutral objects. At the end of each neurofeedback trial, participants rested for 38s, followed by a 4s display of a feedback value and a monetary reward. To promote learning, the threshold for reward was gradually increased across 3 training days so that feedback values were rewarded if they were positive, larger than those on day 2, and larger than those on day 3, respectively. The connectivity-based feedback signal was estimated using Bayesian model comparison[7] between two alternative DCM

model architectures that is a top-down model dominance (from dmPFC onto bilateral amygdala) over a bottom-up one (from bilateral amygdala onto dmPFC)[2]. DCM is a Bayesian framework that models effective connectivity among different brain regions as a set of (ordinary) differential equations[8] and quantitatively tests which model architecture explains the observed data best[7]. The target DCM model represented top-down modulation from dmPFC onto bilateral amygdala and the opposed DCM model represented a bottom-up flow from bilateral amygdala onto dmPFC (**Fig. 1C**). The feedback was provided in terms of the logarithmic Bayes factor, which was positive if the trial was successful, i.e. top-down model was dominating over bottom-up model (for details, see[2]). Neurofeedback was estimated and presented using in-house neurofeedback software that later evolved into the open-source OpenNFT framework[9-11].

Behavioral testing included psychological questionnaires before the experiment and rating positive-social stimuli for valence and arousal before and after the experiment using the self-assessment manikin (SAM) scale[12]. Participants completed the Emotion Regulation Questionnaire (ERQ)[13], Thought Control Ability Questionnaire (TCAQ)[14], White Bear Suppression Inventory (WBSI)[15], State-Trait Anxiety Inventory (STAI)[16], Sensitivity to Punishment and Sensitivity to Reward Questionnaire (SPRSQ)[17,18], and Beck Depression Inventory[19]. None of the participants endorsed depressive symptoms (BDI scores were ~1.5 ± 1.1). Participants also completed the neurofeedback training questionnaire to describe feedback signal manipulations, effective strategies, and attentional demands.

Participants performed pre-/post- transfer runs to test if the learned self-regulation transferred to situations without neurofeedback. The control group underwent the same experimental procedures but received sham feedback derived from feedback values of the best performing participants in the experimental group. Debriefing interviews conducted after the experiment confirmed that control participants were unaware that they had received sham feedback.

We performed a multivariate analysis of variance (MANOVA) to detect a main effect of group (i.e., between experimental and control groups) given three dependent variables, namely, the differences in valence and arousal ratings, and the slopes of the learning curves. A MANOVA can reveal hidden dependencies between the dependent variables that may not be detected with individual testing. To further elucidate the effect of neurofeedback training on subjectively-experienced levels of valence and arousal, we performed two separate analyses of variance (ANOVAs) with the factor group (experimental vs. control) and learning success as a covariate. We justified the equal variances for valence and arousal differences using a F-test of equal variances. We also evaluated the effect of unequal group sizes on

observed differences between experimental and control group behavioral estimates assuming equal group sizes between them. The difference in learning success between the experimental and control group was estimated post-hoc using a two-tailed two-sample t-test. Next, we analyzed the difference in the resulting logarithmic Bayes factors between the pre- and post-training transfer runs, as well as between the participants in the experimental group and those in the control group using one-tailed Wilcoxon rank sum tests and z-statistics. This approach was used because a Jarque-Bera test established that the logarithmic Bayes factors of these runs were not normally distributed. Further details on experiments and neurofeedback task-related and behavioral data analyses have been reported elsewhere[2].

MRI data acquisition

Imaging data were acquired on a 3T whole body MRI system equipped with a 32-channel head receive coil (Trio Tim, Siemens Medical Solutions, Erlangen, Germany). Each scanning session started with the acquisition of a T_1-weighted structural scan (3D MPRAGE, voxel size = 1mm^3 isotropic, flip angle α = 9°, TR = 1900ms, TI = 900ms, TE = 2.27ms), and a double-echo FLASH fieldmap (TE1 = 5.19ms, TE2 = 7.65ms, 3×3×2.2mm^3 voxels). Partial brain coverage fMRI scans for neurofeedback training and transfer runs were acquired with a single-shot gradient-echo T_2*-weighted EPI sequence (1050 and 252 scans for training and transfer runs, TR = 1100ms, TE = 30ms, 18 slices with 25% distance factor, 120×120 matrix, 1.8mm^3 isotropic voxels, flip angle α = 70°, bandwidth = 1.54kHz/pixel, TE = 30ms, GRAPPA with iPAT = 3). This EPI protocol was designed to ensure a precise subdivision of the target prefrontal and limbic brain areas[20,21], and had a short TR to limit the effects of slice timing differences on the DCM[22,23]. All participants were instructed to remain as still as possible and to breathe steadily.

Supplemental References

1 Eickhoff, S. B. *et al.* A new SPM toolbox for combining probabilistic cytoarchitectonic maps and functional imaging data. *Neuroimage* **25**, 1325-1335, doi:DOI 10.1016/j.neuroimage.2004.12.034 (2005).

2 Koush, Y. *et al.* Learning control over emotion networks through connectivity-based neurofeedback. *Cerebral cortex* **27**, 1193-1202 (2017).

3 Sitaram, R. *et al.* Closed-loop brain training: the science of neurofeedback. *Nature Reviews Neuroscience* **18**, 86-100 (2017).

4 Lang, P. J., Greenwald, M. K., Bradley, M. M. & Hamm, A. O. Looking at Pictures - Affective, Facial, Visceral, and Behavioral Reactions. *Psychophysiology* **30**, 261-273, doi:DOI 10.1111/j.1469-8986.1993.tb03352.x (1993).

5 Marchewka, A., Zurawski, L., Jednorog, K. & Grabowska, A. The Nencki Affective Picture System (NAPS): Introduction to a novel, standardized, wide-range, high-quality, realistic picture database. *Behavior research methods*, doi:10.3758/s13428-013-0379-1 (2013).

6 Dan-Glauser, E. S. & Scherer, K. R. The Geneva affective picture database (GAPED): a new 730-picture database focusing on valence and normative significance. *Behavior research methods* **43**, 468-477, doi:DOI 10.3758/s13428-011-0064-1 (2011).

7 Penny, W. D., Stephan, K. E., Mechelli, A. & Friston, K. J. Comparing dynamic causal models. *Neuroimage* **22**, 1157-1172 (2004).

8 Friston, K. J., Harrison, L. & Penny, W. Dynamic causal modelling. *Neuroimage* **19**, 1273-1302 (2003).

9 Koush, Y., Zvyagintsev, M., Dyck, M., Mathiak, K. A. & Mathiak, K. Signal quality and Bayesian signal processing in neurofeedback based on real-time fMRI. *Neuroimage* **59**, 478-489, doi:10.1016/j.neuroimage.2011.07.076 (2012).

10 Koush, Y. *et al.* OpenNFT: An open-source Python/Matlab framework for real-time fMRI neurofeedback training based on activity, connectivity and multivariate pattern analysis. *Neuroimage* **156**, 489-503, doi:10.1016/j.neuroimage.2017.06.039 (2017).

11 Koush, Y. *et al.* Real-time fMRI data for testing OpenNFT functionality. *Data in brief* **14**, 344-347, doi:10.1016/j.dib.2017.07.049 (2017).

12 Lang, P. J., Greenwald, M. K., Bradley, M. M. & Hamm, A. O. Looking at pictures: Affective, facial, visceral, and behavioral reactions. *Psychophysiology* **30**, 261-273 (1993).

13 Gross, J. J. & John, O. P. Individual differences in two emotion regulation processes: implications for affect, relationships, and well-being. *Journal of personality and social psychology* **85**, 348-362 (2003).

14 Luciano, J. V., Algarabel, S., Tomas, J. M. & Martinez, J. L. Development and validation of the thought control ability questionnaire. *Pers Indiv Differ* **38**, 997-1008, doi:DOI 10.1016/j.paid.2004.06.020 (2005).

15 Wegner, D. M. & Zanakos, S. Chronic Thought Suppression. *J Pers* **62**, 615-640, doi:DOI 10.1111/j.1467-6494.1994.tb00311.x (1994).

16 Spielberger, C. D., Gorsuch, R.L., Lushene, R., Vagg, P.R., & Jacobs, G.A. . *Manual for the State-Trait Anxiety Inventory.*, (1983).

17 Torrubia, R., Avila, C., Molto, J. & Caseras, X. The Sensitivity to Punishment and Sensitivity to Reward Questionnaire (SPSRQ) as a measure of Gray's anxiety and impulsivity dimensions. *Pers Indiv Differ* **31**, 837-862, doi:Doi 10.1016/S0191-8869(00)00183-5 (2001).

18 Lardi, C., Billieux, J., d'Acremont, M. & Van der Linden, M. A French adaptation of a short version of the Sensitivity to Punishment and Sensitivity to Reward Questionnaire (SPSRQ). *Pers Indiv Differ* **45**, 722-725, doi:DOI 10.1016/j.paid.2008.07.019 (2008).

19 Beck, A. T., Ward, C. H., Mendelson, M., Mock, J. & Erbaugh, J. An inventory for measuring depression. *Archives of general psychiatry* **4**, 561-571 (1961).

20 Weiskopf, N., Hutton, C., Josephs, O. & Deichmann, R. Optimal EPI parameters for reduction of susceptibility-induced BOLD sensitivity losses: a whole-brain analysis at 3 T and 1.5 T. *Neuroimage* **33**, 493-504 (2006).

21 Weiskopf, N., Hutton, C., Josephs, O., Turner, R. & Deichmann, R. Optimized EPI for fMRI studies of the orbitofrontal cortex: compensation of susceptibility-induced gradients in the readout direction. *Magnetic Resonance Materials in Physics, Biology and Medicine* **20**, 39 (2007).

22 Kiebel, S. J., Klöppel, S., Weiskopf, N. & Friston, K. J. Dynamic causal modeling: a generative model of slice timing in fMRI. *Neuroimage* **34**, 1487-1496 (2007).
23 Koush, Y. *et al.* Connectivity-based neurofeedback: dynamic causal modeling for real-time fMRI. *Neuroimage* **81**, 422-430 (2013).
